# A Plasmid-Encoded FetMP-Fls Iron Uptake System Confers Selective Advantages to *Salmonella enterica* Serovar Typhimurium in Growth under Iron-Restricted Conditions and for Infection of Mammalian Host Cells

**DOI:** 10.3390/microorganisms8050630

**Published:** 2020-04-27

**Authors:** Vanesa García, Ana Herrero-Fresno, Rosaura Rodicio, Alfonso Felipe-López, Ignacio Montero, John E. Olsen, Michael Hensel, María Rosario Rodicio

**Affiliations:** 1 Department of Functional Biology, Section of Microbiology, University of Oviedo, 33006 Oviedo, Spain; vanesag.menendez@usc.es (V.G.); nachomontero@gmail.com (I.M.); 2 Division of Microbiology, University of Osnabrück, 49076 Osnabrück, Germany; alfonso.felipe@cinvestav.mx (A.F.-L.); Michael.Hensel@uni-osnabrueck.de (M.H.); 3 Department of Veterinary and Animal Sciences, Faculty of Health and Medical Sciences, University of Copenhagen, 1870 Frederiksberg, Denmark; ahefr@sund.ku.dk (A.H.-F.); jeo@sund.ku.dk (J.E.O.); 4 Department of Biochemistry and Molecular Biology, University of Oviedo, 33006 Oviedo, Spain; mrosaura@uniovi.es; 5 Translacional Microbiology Group, Health Research Institute of Principado de Asturias, 33011 Oviedo, Spain (ISPA)

**Keywords:** *Salmonella enterica* serovar Typhimurium, high-affinity iron uptake systems, FetMP-Fls system, iron-restrictive conditions, epithelial cells invasion, macrophage intracellular replication

## Abstract

The resistance plasmid pUO-StVR2, derived from virulence plasmid pSLT, is widespread in clinical isolates of *Salmonella enterica* serovar Typhimurium recovered in Spain and other European countries. pUO-StVR2 carries several genes encoding a FetMP-Fls system, which could be involved in iron uptake. We therefore analyzed *S*. Typhimurium LSP 146/02, a clinical strain selected as representative of the isolates carrying the plasmid, and an otherwise isogenic mutant lacking four genes (*fetMP*-*flsDA*) of the *fetMP*-*fls* region. Growth curves and determination of the intracellular iron content under iron-restricted conditions demonstrated that deletion of these genes impairs iron acquisition. Thus, under these conditions, the mutant grew significantly worse than the wild-type strain, its iron content was significantly lower, and it was outcompeted by the wild-type strain in competition assays. Importantly, the strain lacking the *fetMP*-*flsDA* genes was less invasive in cultured epithelial HeLa cells and replicated poorly upon infection of RAW264.7 macrophages. The genes were introduced into *S*. Typhimurium ATCC 14028, which lacks the FetMP-Fls system, and this resulted in increased growth under iron limitation as well as an increased ability to multiply inside macrophages. These findings indicate that the FetMP-Fls iron acquisition system exceeds the benefits conferred by the other high-affinity iron uptake systems carried by ATCC 14028 and LSP 146/02. We proposed that effective iron acquisition by this system in conjunction with antimicrobial resistance encoded from the same plasmid have greatly contributed to the epidemic success of *S*. Typhimurium isolates harboring pUO-StVR2.

## 1. Introduction

Iron is an essential micronutrient required by nearly all bacteria, including non-typhoid serovars of *Salmonella enterica*, which are among the most common food-borne pathogens causing infections in humans [1]. Although iron is only required at very low concentrations (10^-7^ to 10^-5^ M) for physiological processes [2], iron acquisition is challenging, because it is biologically unavailable in most environmental conditions. Ferrous ion (Fe^2+^), the reduced state required for incorporation into most iron-containing proteins, is only stable under anaerobic conditions, or under oxic conditions at low pH. In the presence of oxygen at physiological pH, Fe^2+^ spontaneously oxidizes to Fe^3+^, the ferric form that is extremely insoluble and therefore useless for cellular processes [2,3]. To worsen the problem, Fe^2+^ ions can activate the Fenton reaction leading to generation of hydroxyl radicals, which are highly toxic, resulting in damage to cellular components [3,4]. Mammalian host chelators, such as ferritin, transferrin, and lactoferrin, prevent oxidative reactions and at the same time withhold this metal from pathogens [5,6].

To counteract the extremely low iron availability both in the external environment and within the host, bacterial pathogens like *S. enterica* have developed a variety of high-affinity iron uptake systems. They are based on three main strategies: (i) uptake of ferric iron by secreting siderophores, (ii) sequestration of ferric iron from exogenous siderophores or iron carrier proteins, and (iii) transport of ferrous iron across the cytoplasmic membrane [7]. Under iron shortage, *S. enterica* can synthetize and secrete the catecholate siderophore enterobactin [8,9], and its C-glycosylated derivative known as salmochelin [10,11]. The latter confers a significant growth advantage against competing bacteria in the infected intestine, because it is not recognized by lipocalin-2, an antimicrobial compound produced by epithelial cells and neutrophils during inflammation [12]. In addition to siderophore production, *S. enterica* highjacks Fe^3+^ from ferri-siderophores secreted by other microorganisms, including fungal (ferrichrome and coprogen) or bacterial (ferrioxamines) hydroxamates [13,14]. In contrast to Fe^3+^ that requires active transport to cross the outer membrane, Fe^2+^ can pass by diffusion through porins. Once in the periplasm, at least one Fe^2+^ (Feo), and two Mn^2+^/Fe^2+^ (Sit and MntH) transporters internalize iron across the cytoplasmic membrane into the cytosol of *S. enterica* [15,16,17].

**Figure 1:**
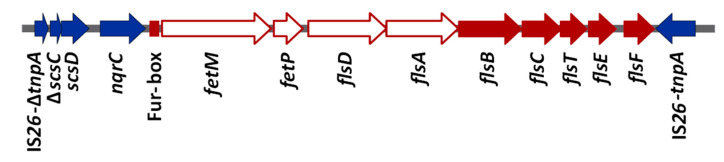
Schematic representation of the *fetMP*-*fls* region of plasmid pUO-StVR2 (flanked by truncated and intact copies of insertion sequence IS*26*). Genes *fetMP*-*flsDA* deleted in the mutant used to investigate the function of the putative FetMP-Fls iron acquisition system are indicated by white arrows; red arrows designate other *fls* genes, the small red square—a putative Fur box, and the blue arrows—additional genes in the *fetMP*-*fls* region. The ORFs from *fetM* to *flsF* were previously designated as ORF-R1 to ORF-R9 [24].

In previous studies, our group characterized an antibiotic resistance-encoding plasmid named pUO-StVR2, which is derived from pSLT, the virulence plasmid of *S. enterica* serovar Typhimurium (*S*. Typhimurium). This plasmid is widespread in clinical isolates from Spain and Portugal and was also detected in strains from the United Kingdom and Italy [18,19,20,21,22]. pUO-StVR2 contains a group of ORFs, here designated the *fetMP*-*fls* cluster (Figure 1), which are transcribed in the same orientation and preceded by a potential binding site for the ferric uptake regulator (Fur), which acts as a transcriptional repressor of genes and operons involved in iron uptake, using Fe^2+^ as a corepressor [2,23].

Previous information together with sequence comparisons suggest that the *fetMP*-*fls* region specifies an iron uptake system [24]; see Section 3.1. In *S. enterica*, homologous genes were only found in *S*. Typhimurium T000240 as part of a new genomic island of 82 kb termed GI-DT12, which also contains all the resistance genes carried by pUO-StVR2 [25], as well as in plasmids of *S*. Enteritidis strain S14 and *S*. Wien strain ZM3 (accession numbers MN328348 and MK797990, respectively). Nevertheless, homologs of this system are detected in annotated genomes of numerous bacteria from different hosts and environments [26], although functional information is only available for a limited number, including strains of human pathogens *Escherichia coli*, *Yersinia pestis*, and *Campylobacter jejuni* [26,27,28,29].

In order to gain evidence on the role of the FetMP-Fls system encoded by pUO-StVR2 in *S. enterica*, a mutant strain of *S*. Typhimurium lacking the *fetM*, *fetP*, *flsD*, and *flsA* genes was generated in the present study. Functional analysis demonstrated that in comparison to the wild-type strain the mutant showed reduced ability to grow under iron-restricted conditions, contained a significantly lower concentration of intracellular iron, and could not efficiently invade epithelial cells or replicate within macrophages.

## 2. Materials and Methods

### 2.1. Bacterial Strains, Plasmids, and Culture Conditions

LSP 146/02, a clinical strain recovered from feces of a 62-year man with gastroenteritis, was chosen as representative of wild-type *S*. Typhimurium isolates carrying plasmid pUO-StVR2 and hence the putative iron uptake system [24]. pUO-StVR2 confers resistance to ampicillin, chloramphenicol, streptomycin/spectinomycin, sulfonamides, and tetracyclines, thus limiting the available selection markers required for generation of recombinant strains (see below). ATCC 14028 reference strain, positive for pSLT, and its mutant derivatives MvP818 (Δ*invC*::FRT impaired in invasion) and P2D6 (*ssaV*::mTn*5* deficient in intracellular replication), were employed as controls in different experiments [30,31]. *E. coli* DH5α (*lacZ*ΔM15, *recA1*, and *endA1* [32]) was used as the host strain for gene cloning. Unless stated otherwise, the strains were routinely grown in a Luria–Bertani (LB; 10 g/L tryptone, 5 g/L yeast extract, and 10 g/L NaCl, pH 7.0–7.4) broth or an LB agar, supplemented with ampicillin (100 µg/mL), kanamycin (50 µg/mL), gentamicin (20 µg/mL), or trimethoprim (10 µg/mL) when required.

### 2.2. In Silico Studies

Whole genome sequencing of *S.* Typhimurium LSP 146/02 harboring pUO-StVR2 was performed with the single-molecule real-time (SMRT) technology of Pacific Biosciences (PacBio, Menlo Park, CA, USA). Briefly, genomic DNA was extracted with a GenElute Bacterial Genomic DNA Kit (Sigma Aldrich, Darmstadt, Germany) and sequenced at Expression Analysis Inc. (Durham, NC, USA) from a library of about 6,500 bp fragments using the PacBio RSII platform. The reads were assembled with the Hierarchical Genome Assembly Process (HGAP 3) version 3.0. The assembly comprised two contigs, one corresponding to the entire chromosome and one to pUO-StVR2, which were annotated by the Prokaryotic Genomes Automatic Annotation Pipeline (PGAAP) of the National Center for Biotechnology Information (NCBI, USA). Genes related to iron uptake were identified by means of a database expressly built for the present study. To update previously obtained information [24], the *fetMP*-*fls* region of pUO-StVR2 (Figure 1) was thoroughly characterized and bacterial homologs were identified with blastn and blastx [33]. The products of the genes were compared with homologs using the multiple sequence alignment tool of Clone Manager (CmSuite9). The sequences are deposited in GenBank under accession numbers CP019950 (chromosome) and CP019951 (pUO-StVR2).

### 2.3. Construction of an S. Typhimurium LSP 146/02 fetMP-flsDA Deficient Strain

To investigate the role of the putative iron uptake system of pUO-StVR2, genes *fetM*, *fetP*, *flsD*, and *flsA* (Figure 1) were deleted from the *S*. Typhimurium LSP 146/02 genome by using the one-step gene inactivation method (previously described by Datsenko and Wanner [34]). The basic strategy of this method is to replace any DNA sequence by a selectable resistance cassette via homologous recombination. In the present study, plasmid pKD13 served as a template for PCR amplification of a kanamycin resistance gene [*aph(3)*-*IIIa*] flanked by FRT (FLP recombinase recognition target) sites [34] using primers containing 40 nt 5′-extensions homologous to the ends (upstream and downstream) of the DNA to be deleted (Table S1). The resulting PCR product was transformed into competent LSP 146/02 cells containing pKD46-Gm [35]. This plasmid has the gentamicin resistance gene *aac(3)*-*Id* as a selectable marker, and carries the *exo*, *bet*, and *gam* genes of the λ-Red recombination system under the control of the inducible *araBAD* promoter. After induction with 10 mM arabinose of the λ-phage proteins in the strain harboring pKD46-Gm, the replacement of the *fetMP*-*flsDA* genes by the kanamycin resistance cassette was verified by PCR amplification using appropriate primers (Table S1). The generated strain, *S*. Typhimurium LSP 146/02 Δ*fetMP*-*flsDA*::*aph(3)*-*IIIa*, was directly employed in competition assays (see below). In addition, the kanamycin resistance cassette was removed by FLP-mediated recombination using plasmid pCP20-Gm that carries gentamicin [*aac(3)*-*Id*] and chloramphenicol (*cat*) resistance genes, and codes for an FLP recombinase which acts on directly repeated FRT sites. The λ-Red and FLP helper plasmids are easily cured by growth at 42 °C because of their temperature-sensitive replicon [35]. The mutant obtained (*S*. Typhimurium LSP 146/02 Δ*fetMP*-*flsDA*::FTR) will be designated Δ*fetMP*-*flsDA* from now on.

### 2.4. Bacterial Growth under Iron-Restricted Conditions

The involvement of the FetMP-Fls system of pUO-StVR2 in iron uptake was first tested by following the growth of LSP 146/02, Δ*fetMP*-*flsDA*, and ATCC 14028 strains in an LB broth containing 0, 10, 50, and 100 µM concentrations of chelating agent 2,2′-dipyridyl (Sigma), or in a PCN minimal medium containing all the micronutrients needed for bacterial growth, except iron. It consisted of 50 mL 10 x MES buffer (800 mM MES, 40 mM tricine, 3.76 mM K_2_SO_4_, 500 mM NaCl); 50 mL 250 mM phosphate buffer, pH 7.4; 5 mL 1.5 M NH_4_Cl; 10 mL 20% glucose; 0.5 mL 1M MgSO_4_ × 7 H_2_O; 0.5 mL 10 mM CaCl_2_ × 2 H_2_O; 0.5 mL 10,000 × micronutrients (10 µM Na_2_MoO_4_ × 2 H_2_O; 10 µM NaSeO_3_ × 5 H_2_O; 4 µM H_3_BO_3_; 0.3 mM CoCl_2_ × 6 H_2_O; 0.1 mM CuSO_4_ × 5 H_2_O; 0.8 mM MnCl_2_ × 4 H_2_O; 0.1 mM ZnSO_4_ x 7 H_2_O); and 384 mL H_2_O_dd_ [36] and was further supplemented with 0.2, 2, 20, or 100 µM of FeCl_3_ (Sigma). Overnight cultures of the *S*. Typhimurium strains were used to inoculate flasks containing 50 mL of each media to an OD_600_ of 0.01, which were incubated with shaking (250 rpm) at 37 °C. Growth was recorded by following the increase in the OD_600_ of the cultures every 2 h for 12 h, and after 24 h.

### 2.5. Intracellular Metal Content Determination by Inductively Coupled Plasma-Mass Spectrometry (ICP-MS)

The total amount of intracellular iron and two other transition metals, manganese and zinc, was quantified by ICP-MS, using a modification of the procedure described in [37]. *S*. Typhimurium LSP 146/02 wild-type and Δ*fetMP*-*flsDA* strains were grown in an LB broth containing 50 µM of 2,2′-dipyridyl to the late logarithmic phase. Cells were harvested by centrifugation at 7000 × g for 7 min and washed three times with 20 mM Tris-HCl, 1 mM EDTA (pH 7.4) followed by three times with 20 mM Tris-HCl (pH 7.4). Bacterial pellets were desiccated at 95 °C overnight to determine the dry cell weight. Pellets were resuspended in 35% HNO_3_ (Trace Metal Grade, Fisher Chemical, Madrid, Spain) and heated at 90 °C for 4 h. After removal of debris by centrifugation, samples were diluted with MQ water to a final concentration of 3.5% HNO_3_. Metal content was determined by ICP-MS on an Agilent 7700 x ICP-MS (Santa Clara, CA, United States) at the Technical Services of the University of Oviedo. Results are expressed in µg of metal/g of cell dry weight.

### 2.6. Competition Assays

To test the ability of the LSP 146/02 wild-type strain to compete for iron against the mutant lacking the *fetMP*-*flsDA* genes, both strains were co-cultured under iron-restricted and non-restricted conditions. In order to differentiate the two strains, LSP 146/02 was labelled with a trimethoprim resistance gene. For this, the *phoN* gene of the wild-type strain, which encodes a non-essential acid phosphatase [38], was replaced by the *dfrA12* gene from plasmid pUO-STmRV1 [39] using the one-step inactivation method as described in Section 2.3 [34] and the primers shown in Table S1. The correct substitution of *phoN* by *dfrA12* was confirmed by PCR amplification using suitable primers (Table S1). LSP 146/02 Δ*phoN*::*dfrA12* and LSP 146/02 Δ*fetMP*-*flsDA*::*aph(3)*-*IIIa* mixed at a 1:1 ratio (1 x 10^7^ CFU/mL each) were grown together at 37 °C with shaking for 10 h in a PCN medium supplemented with 0.2, 2, 20, or 100 mM FeCl_3_. Growth was monitored every 2 h by spreading 100 µl of tenfold serial dilution on an LB agar containing either trimethoprim or kanamycin and quantifying the CFU/mL corresponding to each strain.

### 2.7. Infection of Eukaryotic Cell Lines

The ability of LSP 146/02 and Δ*fetMP*-*flsDA* strains to invade HeLa cells, a classical model for *Salmonella* invasion of epithelial cells, and to replicate within macrophage cell line RAW264.7 (lacking a functional natural resistance-associated macrophage protein 1, Nramp1^-/-^) was tested. Relevant *Salmonella* control strains were included in these experiments (see below). HeLa and RAW264.7 cells (Cell Lines Service (CLS), Heidelberg, Germany) were propagated in a high-glucose (4.5 g/L) Dulbecco’s modified Eagle’s medium (DMEM) containing 4 mM glutamine (PAA, Cölbe, Germany) and 10% (HeLa) or 6% (RAW264.7) inactivated fetal calf serum (iFCS) (Sigma), at 37 °C in an atmosphere of 5% CO_2_. For the infection experiments, cells were seeded in 24-well plates to confluence with approximately 2 × 10^5^ and 4 × 10^5^ cells per well for HeLa cells and RAW264.7, respectively, at the day of infection.

#### 2.7.1. Invasion of HeLa Cells

In these experiments, the *S.* Typhimurium LSP 146/02 wild-type and Δ*fetMP*-*flsDA* strains were tested together with ATCC 14028 and its isogenic Δ*invC*::FRT mutant [30], which were used as positive and negative controls for invasion, respectively. Strains were grown overnight in an LB broth at 37 °C. The cultures were diluted 1:30 with a fresh LB broth and incubated for further 3.5 h at 37 °C with shaking to reach the late logarithmic growth phase to induce expression of invasion genes. Bacteria were added to the HeLa cells at the multiplicity of infection (MOI) of 5. The plates were centrifuged at 500 rpm for 5 min in order to synchronize infection, and incubated for 25 min at 37 °C under 5% CO_2_. After infection, HeLa cells were washed with the PBS and incubated for 1 h in a DMEM supplemented with 100 µg/mL gentamicin to kill free bacteria. HeLa cells were then washed with a pre-warmed PBS and lysed with 0.1% Triton X-100 in the PBS for 10 min at room temperature. Serial dilutions of the lysates were plated onto the Müller–Hinton (MH) agar for quantification of CFU/mL. Rates of invasion are expressed as the percentage of CFU/mL corresponding to gentamicin-protected bacteria with respect to the initial inoculum.

#### 2.7.2. Replication within Macrophages

Together with the *S*. Typhimurium LSP 146/02 wild-type and Δ*fetMP*-*flsDA* strains, the ATCC 14028 strain and the isogenic *ssaV*-mTn*5* derivative [31] were included as positive and negative controls, respectively. All strains were grown overnight in an LB broth with appropriate antimicrobial selection, and RAW264.7 macrophages were infected at the MOI of 1. After 25 min, non-internalized bacteria were removed by washing with PBS and the remaining extracellular bacteria were killed by incubation in a DMEM supplemented with 100 µg/mL of gentamicin for 1 h and then, to quantify intracellular replication, with 10 µg/mL gentamicin for 1 and 15 h (2 and 16 h post-infection). After this treatment, RAW264.7 cells were washed and lysed with 0.1% Triton X-100 in the PBS. Serial dilutions of the lysates were plated onto the MH agar for the quantification of CFU/mL. Rates of intracellular proliferation were calculated as the ratio of gentamicin-protected bacteria recovered from host cells 2 and 16 h post-infection.

### 2.8. Complementation Studies and Transformation of S. Typhimurium ATCC 14028 with the Cloned fetMP-flsDA Genes

For complementation studies, 5,566 bp of pUO-StVR2 containing *fetMP*-*flsDA* and the upstream promoter region were cloned as an XbaI/KpnI fragment into low copy number vector pWKS130, which has the kanamycin resistance *aphA1* gene as a selectable marker [40]. The DNA of pUO-StVR2 was extracted with a PureLink^TM^ HiPure Plasmid DNA Purification Kit (Invitrogen, Madrid, Spain) and used as a template for PCR amplification of the fragment with forward and reverse primers containing recognition sites for XbaI and KpnI at the 5′-end, respectively (Table S1). The PCR product and the pWKS130 vector were digested with these enzymes, mixed, ligated, and transferred into *E. coli* DH5α competent cells [41]. A plasmid carrying the right insert named pWKS130::*fetMP*-*flsDA* was introduced into LSP 146/02 Δ*fetMP*-*flsDA* and also into *S*. Typhimurium ATCC 14028 to investigate the effect of the iron uptake system of pUO-StVR2 on this bacterium. The generated strains (LSP 146/02 Δ*fetMP*-*flsDA*^C^ and ATCC 14028 *fetMP*-*fls*^T^) were tested for growth under iron-limiting and non-limiting conditions, invasion of HeLa cells, and replication within macrophages as described above.

### 2.9. Statistical Analysis

Results of all the assays carried out in this work are expressed as the mean values ± standard deviations (SD) of three biological replicates. Statistical analysis was performed with the GraphPad Prism version 8.3 software (GraphPad Inc). For the competition assays, CFU data were log10 transformed and compared at each time point tested. For analysis of the results related to infection of cell lines, raw CFU/mL data were used and % of invasion or fold net replication were determined and compared. Statistical significance was established using a one-sample *t*-test analysis (for intracellular metal content and competition assays) or a one-way ANOVA with the Tukey’s multiple comparison post-test (for growth curves based on OD measurements and infection of cell lines). *p*-values < 0.05 were considered significant.

## 3. Results

### 3.1. Multiple High-Affinity Iron-Uptake Systems are Encoded by the Genome of S. Typhimurium LSP 146/02

Sequence analysis of the genome of *S*. Typhimurium LSP 146/02 revealed the presence of genes encoding numerous systems involved in iron uptake, including i) the genes required for production and secretion of siderophores enterobactin and salmochelin, import of the Fe^3+^-loaded siderophores, and cytoplasmic reduction of Fe^3+^ to Fe^2+^ for incorporation into bacterial proteins; ii) the genes for uptake of exogenous ferri-siderophores (ferrichrome, rhodotorulic acid, and ferrioxamine); and iii) the genes for production of Fe^2+^ transporters Feo, Sit, and MntH (Table S2). These high-affinity iron uptake systems are widely spread and highly conserved in serovars and strains of *S. enterica*, including *S*. Typhimurium LT2 and ATCC 14028. Comparison of the LSP 146/02 regions encoding these systems revealed that they are identical to homologous regions in the genome of LT2 and nearly identical to those in ATCC 14028 (Table S3). Thus, they are likely to be functional in LSP 146/02.

Besides the former systems, plasmid pUO-StVR2 provides to *S*. Typhimurium LSP 146/02 the *fetMP*-*fls* genes, previously designated ORF-R1 to ORF-R9 (Figure 1), which could specify an additional iron uptake system [24]. Sequence analysis identified the product of ORF-R1 as a membrane protein belonging to a subgroup of the iron/lead transporter (ILT) superfamily homologous to the FetM protein of uropathogenic *E. coli* strain F11, whereas the product of ORF-R2 resembles a periplasmic iron binding protein related to the *E. coli* F11 FetP that, together with FetM, constitutes an Fe^2 +^ /Fe^3+^ uptake system regulated by the Fur [27]. The ORFs downstream of the *fetMP*-like genes (ORF-R3 to ORF-R9), here termed *fls* (for *fet*-linked *Salmonella*), were predicted to encode a membrane protein of unknown function (*flsD*); a previously uncharacterized ABC transporter with two permease components (*flsA* and *flsB*) and an ATPase component (*flsC*); and a periplasmic protein of the thioredoxin-like (TRL) superfamily (*flsT*). The products of these genes are identical or highly similar to products of the equivalent genes found in *S*. Typhimurium T000250, *S*. Enteritidis S14, *S*. Wien ZM3, and *E. coli* F11, and more distantly related to those of *Y. pestis* KIM5 (identities ranging from 56% to 88%) and *C. jejuni* 81-176 (from 27% to 57%) (Table S4; [25,26,27,28,29]). *flsE* and *flsF*, which specify a hypothetical protein and a member of the histidine phosphatase (HP) superfamily, are not conserved in *fetMP*-*fls*-like clusters.

### 3.2. Growth of the S. Typhimurium LSP 146/02 ΔfetMP-flsDA Strain is Impaired under Iron-Restrictive Conditions

In order to assess the physiological contribution of the *fetMP*-*fls* cluster to iron acquisition, growth of wild-type strain LSP 146/02 and its isogenic *fetMP*-*flsDA* deletion mutant were compared under iron-restrictive growth conditions. *S*. Typhimurium ATCC 14028, usually employed for virulence studies in *Salmonella*, was included as a control.

**Figure 2:**
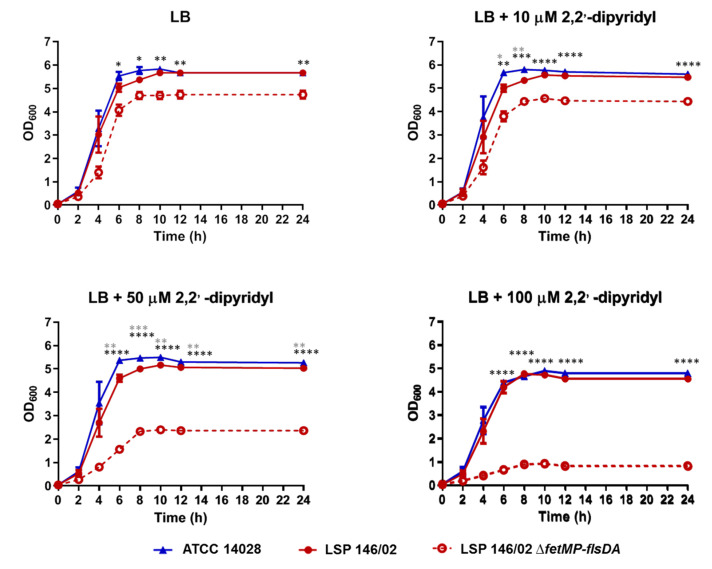
Growth curves obtained for *S*. Typhimurium ATCC 14028, wild-type *S.* Typhimurium LSP 146/02, and its isogenic Δ*fetMP*-*flsDA* derivative using an LB broth supplemented with increasing concentrations of chelating agent 2,2′-dipyridyl. The data shown are the mean values ± standard deviations of three biological replicates. Statistical significance (**** *p*  <  0.0001; *** *p*  <  0.001; ** *p*  <  0.01; * *p*  <  0.05) was determined by ANOVA and the Tukey’s post-test. Black and grey asterisks show significant differences for LSP 146/02 versus Δ*fetMP*-*flsDA* and ATCC 14028, respectively.

**Figure 3:**
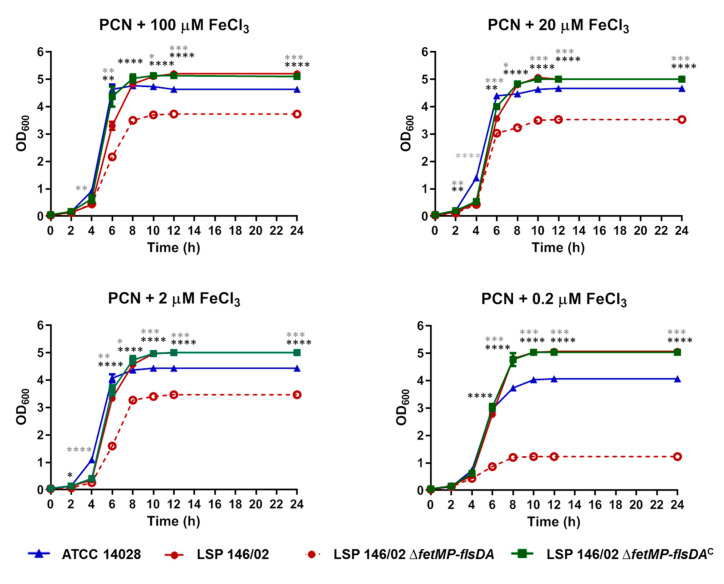
Growth curves obtained for *S*. Typhimurium ATCC 14028, *S.* Typhimurium LSP 146/02 wild-type, its isogenic Δ*fetMP*-*flsDA* derivative, and the complemented LSP 146/02 Δ*fetMP*-*flsDA*^C^ strain using a PCN medium supplemented with decreasing concentrations of FeCl_3_. The data shown are the mean values ± standard deviations of three biological replicates. Statistical significance (**** *p*  <  0.0001; *** *p*  <  0.001; ** *p*  <  0.01; * *p*  <  0.05) was determined by ANOVA and the Tukey’s post-test. Black and grey asterisks show significant differences for LSP 146/02 versus Δ*fetMP*-*flsDA* and ATCC 14028, respectively. Significant differences were not observed for LSP 146/02 versus LSP 146/02 Δ*fetMP*-*flsDA*^C^.

As shown in Figure 2, 2,2′-dipyridyl slightly affected the growth of both LSP 146/02 and ATCC 14028 at concentrations of 10 and 50 µM, while bacterial growth decreased by approximately 17% in the presence of 100 µM of the chelating agent. Interestingly, growth of the Δ*fetMP*-*flsDA* strain was severely impaired at 50 µM (55% reduction) and 100 µM (83% reduction) of 2,2′-dipyridyl, despite the presence of the many other iron uptake systems expected to be functional according to sequence comparisons. In an iron-free PCN medium supplemented with decreasing concentrations of FeCl_3_, growth of the mutant was reduced by approximately 30% compared to the isogenic wild-type strain at 100, 20, or 2 µM FeCl_3_, while bacteria barely grew at 0.2 µM FeCl_3_ (80% reduction; Figure 3). Complementation Δ*fetMP*-*flsDA* with a low copy number vector harboring the *fetMP*-*flsDA* genes under the control of the native promoter region restored the ability of the mutant to grow like the wild-type strain under iron-limiting conditions (Figure 3).

### 3.3. The Intracellular Iron Content is Higher in S. Typhimurium LSP 146/02 than in the ΔfetMP-flsDA Mutant

The growth behavior of Δ*fetMP*-*flsDA* on iron-restricted media indicates an important role of the *fetMP*-*fls* system in iron uptake. This was further corroborated by comparison of the intracellular iron concentrations in LSP 146/02 and its isogenic mutant, which still carries the genes for many other iron uptake systems.

As shown in Table 1, the iron concentration was significantly lower (*p* ˂ 0.0005) in the mutant than in the wild-type strain. In contrast, no significant differences were observed for manganese, an iron analogue with shared transport systems, like SitABCD and MntH [17], or zinc, the second most abundant transition metal in living organisms [42].

### 3.4. S. Typhimurium LSP 146/02 Outcompetes its ΔfetMP-flsDA Mutant Strain under Iron Limitation

**Figure 4:**
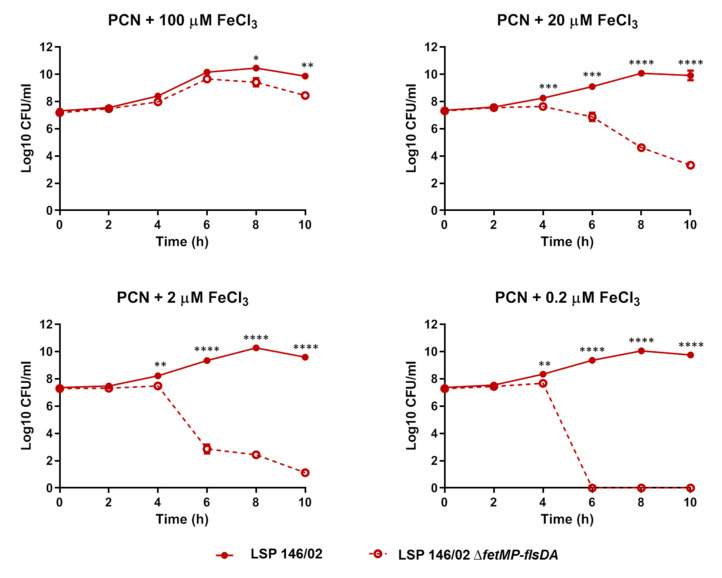
Competition assays of the *S.* Typhimurium LSP 146/02 wild-type strain and its isogenic derivative Δ*fetMP*-*flsDA* using a PCN medium with different concentrations of FeCl_3_. The data shown are the mean values ± standard deviations of three biological replicates. Statistical significance (**** *p* <  0.0001; *** *p* <  0.001; ** *p* <  0.01; * *p*  <  0.05) was determined using a one-sample *t*-test at each time point tested.

Competition assays were performed to compare the fitness of the Δ*fetMP*-*flsDA* strain relative to wild-type LSP 146/02 under iron-limiting conditions. To distinguish the strains, the wild-type and the mutant labelled with trimethoprim (LSP 146/02::*dfrA12*) and kanamycin [LSP 146/02 Δ*fetMP*-*flsDA*::*aph(3)*-*IIIa*] resistance cassettes, respectively, were employed. The results are shown in Figure 4. In accordance with the role of the deleted ORFs in iron uptake, the mutant strain was totally outcompeted by the wild-type strain 6 h post-inoculation in a PCN medium supplemented with the lowest iron concentration (0.2 µM). The same result was obtained 10 h post-inoculation at 2 µM, and displacement of the mutant by the wild-type strain was still observed at higher iron concentrations (with significant differences detected at t = 8 h and t = 10 h post-inoculation).

### 3.5. Invasion of Epithelial Cells and Replication within Macrophages Decrease in the Absence of the fetMP-flsDA Genes

**Figure 5:**
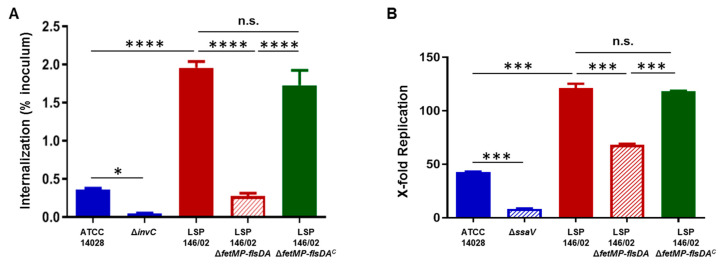
Invasion of epithelial HeLa cells (**A**) and replication within RAW264.7 macrophages (**B**) by wild-type *S.* Typhimurium LSP 146/02, its Δ*fetMP*-*flsDA* isogenic derivative, and the latter strain complemented with the cloned *fetMP*-*flsDA* genes (LSP 146/02 Δ*fetMP*-*flsDA*^C^). The data shown are the mean values ± standard deviations of three biological replicates. Statistical significance (**** *p*  <  0.0001; *** *p*  <  0.001; ** *p*  <  0.01; * *p*  <  0.05; n.s., not significant) was determined by one-way ANOVA and the Tukey´s post-test.

In order to assess the role of the iron uptake system in virulence, the invasion of epithelial HeLa cells by the wild-type LSP 146/02 and Δ*fetMP*-*flsDA* strains was investigated. ATCC 14028 and its isogenic *invC* deficient mutant were included as positive and negative controls, respectively. As shown in Figure 5A, the mutant strain lacking the iron uptake system was significantly less invasive (7-fold reduction) than LSP 146/02 (*p* < 0.0001). The same strains were tested for the ability to replicate intracellularly within RAW264.7 macrophages, except for the negative control, for which the *ssaV* mutant of ATCC 14028 instead of the *invC* mutant was employed. Proliferation of Δ*fetMP*-*flsDA* was significantly reduced (1.7-fold) when compared to LSP 146/02 (*p* < 0.0001) (Figure 5B). Moreover, we confirmed that introduction of the intact *fetMP*-*flsDA* genes restored the ability of the mutant strain to invade HeLa cells and to replicate within macrophages, reaching wild-type levels (Figure 5A,B).

### 3.6. The FetMP-Fls System of pUO-StVR2 Increases Performance of S. Typhimurium ATCC 14028 under Iron-Limiting Conditions

**Figure 6:**
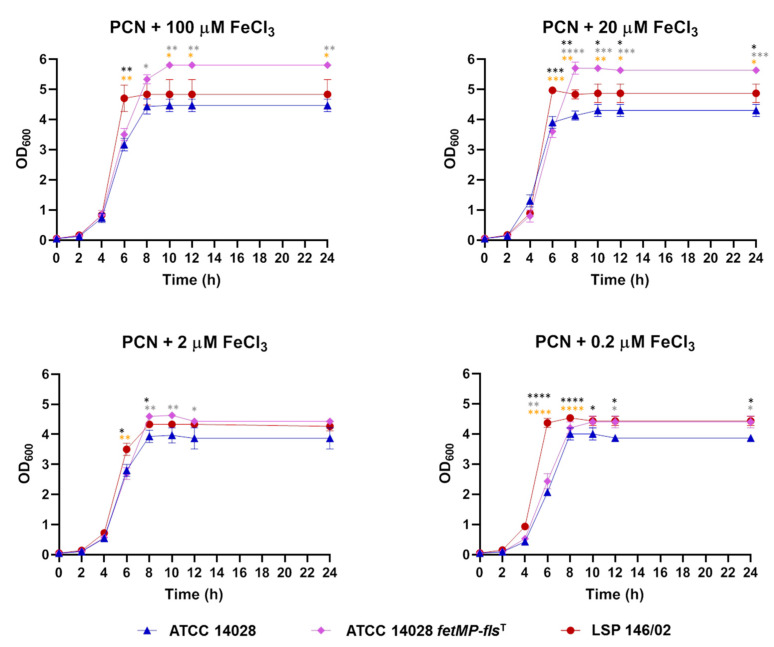
Growth curves obtained for *S*. Typhimurium ATCC 14028 with and without the cloned *fetMP*-*flsDA* genes (ATCC 14028 *fetMP*-*fls*DA^T^) using a PCN medium supplemented with decreasing concentrations of FeCl_3_. The data shown are the mean values ± standard deviations of three biological replicates. Statistical significance (**** *p*  <  0.0001; *** *p*  <  0.001; ** *p*  <  0.01; * *p*  <  0.05) was determined by ANOVA and the Tukey’s post-test. Black, grey, and orange asterisks show significant differences for ATCC 14028 versus LSP 146/02, ATCC 14028 versus ATCC 14028^T^, and LSP 146/02 versus ATCC 14028 *fetMP*-*flsDA*^T^, respectively.

**Figure 7:**
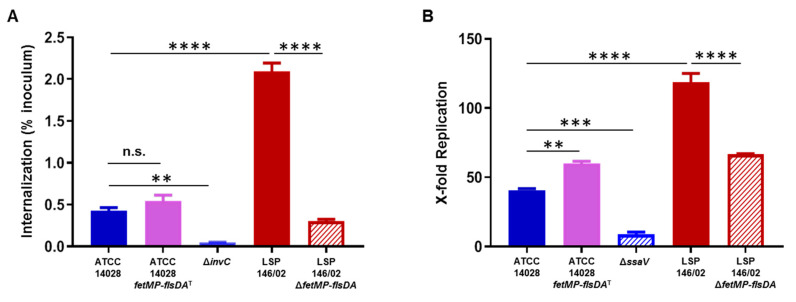
Invasion of epithelial HeLa cells by *S.* Typhimurium ATCC 14028 harboring the cloned *fetMP*-*flsDA* genes (ATCC 14028 *fetMP*-*flsDA*^T^) (**A**) and replication of the same strain within RAW264.7 macrophages (**B**). The data are the mean values ± standard deviations of three biological replicates. Statistical significance (**** *p*  <  0.0001; *** *p*  <  0.001; ** *p*  <  0.01) was determined by one-way ANOVA and the Tukey´s post-test.

To evaluate whether the iron acquisition system encoded by pUO-StVR2 could also provide a selective advantage to *S*. Typhimurium ATCC 14028, the cloned *fetMP*-*flsDA* genes were introduced into this strain and then growth under iron-restricted conditions, invasion of HeLa cells, and replication within RAW264.7 cells were tested. As shown in Figure 6, the transformed strain grew better than the original strain in an iron-free PCN medium with all FeCl_3_ concentrations tested, and it grew similarly or even better than LSP 146/02 depending on the iron concentration in the medium. The cloned DNA also increased the ability of ATCC 14028 to replicate inside macrophages, although it did not improve its capacity to invade epithelial cells (Figure 7).

## 4. Discussion

Taken together, the results presented herein demonstrated that the FetMP-Fls system of pUO-StVR2 is involved in iron uptake and confers benefits to bacteria both under culture and infective conditions. This conclusion is based on three observations: i) when iron was restricted, deletion of the *fetMP*-*flsDA* genes was detrimental to growth and the wild-type strain fully outcompeted the deletion mutant strain; ii) the intracellular iron concentration was five times higher in the presence than in the absence of these genes; and iii) their loss compromised the infection of mammalian host cells by *Salmonella*.

While previous studies shed light on the role of the FetM and FetP proteins in iron uptake, the function of the Fls proteins remains to be established. In the uropathogenic F11 strain of *E. coli*, a mutant deficient in most iron transport systems was used to investigate the role of FetM and FetP [27]. Deletion of *fetM* in this genetic background impaired iron acquisition, whereas *fetP* proved to be dispensable, although required for optimal growth at low Fe^2+^ concentrations and also in the presence of Fe^3+^. Koch et al. [27] proposed that FetM acts as a functional Fe^2+^ transporter and attributed to FetP a dual role as a periplasmic Fe^2+^ binding protein and as a Fe^3+^ reductase. In the present study, growth experiments were performed with decreasing concentrations of FeCl_3_. However, as suggested for the FetMP system of *E. coli* F11 [27], a nearly identical system to the FetMP system of *S*. Typhimurium LSP 146/02, the latter may be equally able to use both oxidative states of iron with or without the cooperation of FetP.

In contrast to *E. coli*, the *fetP* gene of *Y. pestis*, but not *fepM*, was required for optimal growth under iron limitation [29]. This was also the case for the *fetP* homolog of *C. jejuni* 81-176, termed p19, but the possible involvement of *fetM* in iron uptake was not investigated in the latter bacterium [28]. Like in *E. coli*, experiments in *Y. pestis* were performed with a mutant lacking other iron acquisition systems. In contrast, many other systems were present in the Δp19 (Δ*fetP*) mutant of *C. jejuni* as well as in the Δ*fetMP*-*flsDA* derivative of LSP 146/02 according to an in silico analysis. Therefore, the benefits associated with the FetMP-Fls system of LSP 146/02, and also with the *fetMP*-*flsDA* genes once transferred into *S*. Typhimurium ATCC 14028, appear to exceed those conferred by the many other iron uptake systems found in the two strains The predominant role of FetMP-Fls as an iron scavenger is also supported by the more than 5-fold increase in the intracellular iron content in LSP 146/02 versus the isogenic Δ*fetMP*-*flsDA* derivative under iron-restricted conditions. It has been reported that catecholamine dopamine promotes growth of *S*. Typhimurium both in culture and within bone marrow-derived macrophages, which depends on an increased iron uptake [43]. Dopamine achieves this by at least two mechanisms: i) acting as a pseudo-siderophore that binds iron directly, and ii) increasing the expression of iron uptake genes, particularly *sitABCD* [43]. Similarly, although the existence of a new regulatory mechanism hindering the expression of other iron uptake systems cannot be disregarded, the enhanced iron availability might be enough to account for the selective advantages conferred by an additional FetMP-Fls system to *S*. Typhimurium.

Surprisingly, the Δ*fetMP*-*flsDA* strain grew worse than the wild-type LSP 146/02 strain even in the presence of ample iron (i.e., in an LB broth without 2,2′-dipyridyl as well as in a PCN medium containing FeCl_3_ 100 µM). This suggests that the FetMP-Fls system provides further benefit(s) to the host strain in addition to iron acquisition. They cannot be caused by an enhanced uptake of zinc and manganese, since no significant differences were observed in the intracellular concentrations of these elements between the mutant and the wild-type strain. However, the involvement of the FetMP-Fls system in enhanced transport of other metals cannot be ruled out, particularly since the role of the FlsABC transporter remains unknown.

Although some information is available on the impact of FetMP-Fls-like systems during growth of bacterial pathogens under iron-restrictive culture conditions, their role under infective conditions has not been investigated, until now. Epithelial cells and macrophages are commonly used in virulence studies of facultative intracellular pathogens like *S. enterica*, which can invade, survive, and replicate within host cells [44]. In the present study, the effect of deletion of *fetMP*-*flsDA* on replication of *S*. Typhimurium LSP 146/02 inside HeLa cells was not determined, but the absence of the iron uptake system negatively affected the process of invasion. Deletion of the system also resulted in decreased bacterial replication within RAW264.7 macrophages, consistent with the notion that iron is limited in both cell types and with its efficient acquisition by the bacterial FetMP-Fls system. Boyer et al. [17] investigated the role of the Feo, Sit, and MntH proteins using mutants of a clinical strain of *S*. Typhimurium lacking either one, two, or all three systems and found that Sit was required for replication inside RAW264.7 cells. However, these experiments were performed with macrophages treated with IFN-γ, and even though the differences were detected only after addition of 2,2′-dipyridyl, which can permeate the macrophage membrane and sequester the intracellular Fe^2+^ [17]. In the present study, a significant reduction in replication of the Δ*fetMP*-*flsDA* strain with respect to the wild-type strain was observed, although RAW264.7 macrophages (Nramp1^-/-^) are not particularly efficient in withholding intracellular metals from pathogens, and they were not treated with IFN-γ or 2,2′-dypirydil. A significantly higher replication rate was also observed for ATCC 14028 harboring the *fetMP*-*flsDA* genes, in agreement with enhanced iron acquisition by the FetMP-Fls system.

## 5. Conclusions

The present study reveals the great importance of the FetMP-Fls system in iron acquisition by *S*. Typhimurium LSP 146/02 and ATCC 14028, addressing for the first time the role of one of these systems during infection of epithelial and phagocytic host cells. As it was proposed for the uropathogenic *E. coli* F11 strain [36], the versatility of the FetMP proteins connected with the ability to use both Fe^2+^ and Fe^3+^ would represent an important advantage with respect to the systems specialized on the uptake of just one of the two oxidation states. Accordingly, the highly efficient FetMP-Fls system together with the multiple resistance genes harbored by pUO-StVR2 may have greatly contributed to the successful dissemination of *S*. Typhimurium isolates carrying the plasmid.

## Figures and Tables

**Table 1:** Intracellular metal concentration in *S.* Typhimurium LSP 146/02 and its Δ*fetMP*-*flsAB* mutant.

Strain	Fe [µg/g dw]	Mn [µg/g dw]	Zn [µg/g dw]
LSP 146/02	1516.48 ± 184.32	39.58 ± 9.96	60.05 ± 3.69
LSP 146/02 Δ*fetMP*-*flsDA*	280.69 ± 90.68	31.78 ± 3.89	60.22 ± 7.72
***p*** **-value**	˂ 0.0005	0.2747	0.9742

Intracellular concentrations of iron, manganese, and zinc were determined by inductively coupled plasma-mass spectrometry (ICP-MS) in the cells grown on an LB broth containing 50 µM of 2,2′-dipyridyl to the late logarithmic phase. The results expressed in µg of metal per g of cell dry weight (dw) are the mean values ± standard deviations of three biological replicates, each with three technical replicates. Statistical significance was calculated using a one-sample *t*-test; *p*-values < 0.05 are considered significant.

## Supplementary Materials

The following are available online at https://www.mdpi.com/2076-2607/8/5/630/s1, Table S1. Primers used in this study; Table S2. Genes associated with iron acquisition found in the genome of *Salmonella enterica* serovar Typhimurium LSP 146/02; Table S3. Comparison of iron acquisition regions found in the genomes of *Salmonella enterica* serovar Typhimurium LSP 146/02, ATCC 14028, and LT2; Table S4. Homologs of the FetMP-Fls system of *Salmonella enterica* serovar Typhimurium LSP 146/02.
