# Supplementary material for: A Plasmid-Encoded FetMP-Fls Iron Uptake System Confers Selective Advantages to Salmonella enterica Serovar Typhimurium in Growth under Iron-Restricted Conditions and for Infection of Mammalian Host Cells
Source: Microorganisms. 2020 Apr 27;8(5):630. doi: 10.3390/microorganisms8050630 (PMC7285068; doi:10.3390/microorganisms8050630)
Supplement: Supplementary file 1 [file microorganisms-08-00630-s001.pdf]

Table S1. Primers used in this study

Primer	Sequence (5'-3')	Use for:
ORFR1-Δ13-λ-Red-Fw	*ATCATTTGTATTCTTTGGCGGTGTGCACGTGCGTAACTTC*ATTC CGGGGATCCGTCGACC	Replacement of *fetMP-flsDA* by a kanamycin resistance cassette to generate the Δ*fetMP-flsDA::aph(3)-IIIa* strain
ORFR4-Δ13-λ-Red-Rv	*GTTTAGCGGCCATACAGCACCTCCACAGGATACAGCCGGG*TG TAGGCTGGAGCTGCTTCG	Replacement of *fetMP-flsDA* by a kanamycin resistance cassette to generate the Δ*fetMP-flsDA::aph(3)-IIIa* strain
nqrC-Fw	ACGGTATTTCCGGTGCGACGTTG	Verification of LSP 146/02 Δ*fetMP-flsDA*:: *aph(3)-IIIa*
orf86-Rv	ACCTGGCCGACATCGTTAC	Verification of LSP 146/02 Δ*fetMP-flsDA*:: *aph(3)-IIIa*
K1	CAGTCATAGCCGAATAGCCT	Verification of LSP 146/02 Δ*fetMP-flsDA*:: *aph(3)-IIIa*
K2	CGGTGCCCTGAATGAACTGC	Verification of LSP 146/02 Δ*fetMP-flsDA*:: *aph(3)-IIIa*
phoN-dfrA12-λ-Red-Fw	*GTTTGCGGGAAGACTTTCACCTTCAGTAATTAAGTTTGGG*CGC CAGCCAGGACAGAAATG	Amplification of a trimethoprim resistance cassette to generate LSP 146/02 Δ*phoN::dfrA12*
phoN-dfrA12-λ-Red-Rv	*GCCTGATCCGGAGTGAGTCTTTATGAAAAGTCGTTATTTA*GGG TTTCAGTGGTGCTAACG	Amplification of a trimethoprim resistance cassette to generate LSP 146/02 Δ*phoN::dfrA12*
phoN-Check-Fw	ACCTAATGCGTGTCAGTCAG	Verification of LSP 146/02 Δ*phoN::dfrA12* labelling
phoN-Check-Rv	GGCCAGCCTGATCCGGAGTG	Verification of LSP 146/02 Δ*phoN::dfrA12* labelling
dfrA12-Fw	ACTCGGAATCAGTACGCA	Verification of LSP 146/02 Δ*phoN::dfrA12* labelling
dfrA12-Rv	GTGTACGGAATTACAGCT	Verification of LSP 146/02 Δ*phoN::dfrA12* labelling
ORFR1-Fw-XbaI	GGC**TCTAGA**GTCTTGCCAGCGATCGAAATAACC	Cloning of *fetMP-flsDA* into pWSK130
ORFR5-Rv-KpnI	CGG**GGTACC**AGCCAGAGCATGGTACGTTTAG	Cloning of *fetMP-flsDA* into pWSK130

Fw, forward; Rv, reverse.

Nucleotides in italics indicate the homologous selected target region in the genome of *Salmonella enterica* serovar Tyhimurium LSP 146/02.

Underlined nucleotides represent sequences annealing with pKD13 [1] for *aph(3)-IIIa* amplification or with pUO-STmRV1 [2] for *dfrA12* amplification. Nucleotides in bold indicate the recognition sites for restriction enzymes.

1. Datsenko, K.A.; Wanner, B.L. One-step inactivation of chromosomal genes in *Escherichia coli* K-12 using PCR products. *Proc Natl Acad Sci* **2000**;*97*(12):6640-5.

2. García, P.; Guerra, B.; Bances, M., Mendoza, M.C.; Rodicio, M.R. IncA/C plasmids mediate antimicrobial resistance linked to virulence genes in the Spanish clone of the emerging *Salmonella enterica* serotype 4,[5],12:i. *J Antimicrob Chemother* **2011**;*66*(3):543-9.

Table S2. Genes involved in iron acquisition found in the genome of *Salmonella enterica* serovar Typhimurium LSP 146/02

Gene(s)	Function
**Ferric iron**	
**Catecholate siderophores**	
entABCDEFS	Enterobactin biosynthetic cluster and exporter (EntS)
fepA	Ferric-enterobactin outer-membrane receptor
fepB	Ferric-enterobactin transporter periplasmic protein
fepE	Ferric-enterobactin transport protein
fepG	Ferric-enterobactin transporter permease
fepD	Ferric-enterobactin transporter membrane protein
fepC	Ferric enterobactin transporter ATP-binding protein
fes	Cytosolic ferric-enterobactin esterase
iroB	Glycosil transferase for salmochelin production
iroC	ABC transporter for export of salmochelin
iroD	Cytosolic esterase of ferri-salmochelin
iroE	Periplasmic hydrolase
iroN	Salmochelin receptor
cirA	Catecholate siderophore receptor
**Hydroxamate siderophores**	
foxA	Ferrioxamines receptor
fhuA	Ferrichrome receptor
fhuD	Ferric-hydroxamate transporter substrate-binding subunit-periplasmic
fhuB	Ferric-hydroxamate transporter permease subunit/permease component of the FhuBC ABC transporter
fhuC	Ferri-hydroxamate transporter ATP-binding subunit
fhuE	Coprogen receptor
fhuF	Cytosolic reductase of ferri-hidroxamates/ferric hydroxamate transport ferric iron reductase
**Energy output for transport**	
tonB, *exbB*, *exbD*	Energy output for outer membrane transport of ferric-siderophores
**Ferrous iron**	
feoABC	FeoABC transporter of ferrous iron
sitABCD	SitABCD transporter of Mn^{2+} and Fe^{2+}
mntH	MntH transporter of Mn^{2+} and Fe^{2+}
**Ferrous and ferric iron**	
fetMP	FetMP-like system, with a membrane-bound ferrous iron transporter (FetM) and a periplasmic protein (FetP)
flsD	Predicted membrane protein
flsABC	Components of a new ABC transporter, with two permeases (FlsA and FlsB) and an ATP-binding protein (FlsC)
flsT	Thioredoxin-like protein

S. Typhimurium LSP 146/02 accession numbers: CP019950 (chromosome) and CP019951 (pUO-StVR2).
All except the *fetMP-fls* genes are located on the chromosome.

Table S3. Comparison of iron acquisition regions found in the genomes of *Salmonella enterica* serovar Typhimurium LSP 146/02, ATCC 14028 and LT2

**ATCC 14028 DNA region** position (bp)[a] gene(s) (bp[b])	**LSP 146/02** position (bp) **LT2** position (bp)	**LSP 146/02 *vs* ATCC 14028**[c] nt differences[d] (→ aa change)	**LSP 146/02 *vs* LT2**[c]
**Ent-Fep region** 640308-661786 (21479)[a]	3309659-3331137c 639614-661092	5 nt[d]	100% ID
entD (705c)		100% ID	100% ID
fepA (2256c)		1nt (Ala20Thr)	100% ID
hp (132c)			
fes (1215c)		100% ID	100% ID
ybdZ (219)			
entF (3885)		100% ID	100% ID
fepE (1137)		1 nt (His359Arg)	100% ID
fepC (795c)		100% ID	100% ID
fepG (990c)		100% ID	100% ID
fepD (1008c)		100% ID	100% ID
ybdA (1245)			
fepB (957c)		100% ID	100% ID
entC (1176)		100% ID	100% ID
entE (1611)		100% ID	100% ID
entB (858)		100% ID	100% ID
entA (756)		2 nt (G345T; C469T)	100% ID
**Iro region** 2936231-2946862 (10632 bp)[a]	1090905-1101536c 2915996-2926627	2 nt[d]	100% ID
iroB (1116)		100% ID	100% ID
iroC (3654)		1nt (Asp1188Glu)	100% ID
iroD (1245)		100% ID	100% ID
iroE (936)		100% ID	100% ID
iroN (2181c)		1 nt (Ala154Val)	100% ID
**CirA region** 2347717-2350601 (2885 bp)[a]	1713768-1716653c 2296163-2299048	1 nt[d]	100% ID
cirA (1992c)		100% ID	100% ID
**FoxA region** 412776-415660 (2885)[a]	3555791-3558675c 412083-414967	1 nt[d]	100% ID
foxA (2109)		1 nt (Leu249Pro)	100% ID
**FhuACDB region** 224151-230860 (6710)[a]	3760488-3767197c 223446-230155	2 nt[d]	100% ID
fhuA (2190)		100% ID	100% ID
fhuC (798)		100% ID	100% ID
fhuD (891)		100% ID	100% ID
fhuB (2058)		1 nt (Met132Thr)	100% ID
**FhuE region** 1245637-1248660 (3024)[a]	2721057-2724080c 1287474-1290497	2 nt[d]	100% ID
fhuE (2175c)		2 nt (Glu112Asp; Thr342Ala)	100% ID
**FhuF region** 4820360-4822459 (2100)[a]	4038453-4040552c 4807527-4809626	100% ID	100% ID
fhuF (789c)		100% ID	100% ID
**TonB region** 1841713-1843197 (1485)[a]	2180313-2181797c 1831024-1832508	100% ID	100% ID
tonB (852c)		100% ID	100% ID
**ExbBD region** 3337377-3339560 (2184)[a]	696075-698258c 3319126-3321309	100% ID	100% ID
exbB (735c)		100% ID	100% ID
exbD (426c)		100% ID	100% ID

**Fur region**<br>756616-757820 (1205)[a]	3213428-3214632c<br>756119-757323	100% ID	100% ID
fur (453)		100% ID	100% ID
**FeoABC region**<br>3677352-3680835 (3484)[a]	349730-353213c<br>3663454-3666937	100% ID	100% ID
feoA (228)		100% ID	100% ID
feoB (2319)		100% ID	100% ID
feoC (237)		100% ID	100% ID
**SitABCD region**<br>3026342-3030206 (3865)[a]	1007422-1011286c<br>3006104-3009968	100% ID	100% ID
sitA (918)		100% ID	100% ID
sitB (822)		100% ID	100% ID
sitC (861)		100% ID	100% ID
sitD (849)		100% ID	100% ID
**MntH region**<br>2574771-2577570 (2800)[a]	1486021-1488820c<br>2523219-2526018	100% ID	100% ID
mntH (1242c)		100% ID	100% ID

[a], Genes, intergenic DNA and at least part of the flanking upstream and downstream *orfs* were compared.

[b], Genes located on the complementary strand (c) are indicated.

[c], 100% coverage was obtained for all performed comparisons.

[d], nucleotide (nt) differences are indicated when 100% identity (ID) was not obtained; nt differences other than those leading to amino acid (aa) substitutions were located in the intergenic region between *entF* and *fepE*, upstream of *cirA* and downstream of *fhuF*.

Accession numbers: CP001363, *S*. Typhimurium ATCC 14028; CP019950, *S*. Typhymurium LSP 146/02; NC_003197, *S*. Typhimurium LT2.

Table S4. Homologs of the FetMP-Fls system of *Salmonella enterica* serovar Typhimurium LSP 146/02

		% Identity[a]							
**Bacteria (plasmid)**	**Gene names in data bases**	**FetM**	**FetP**	**FlsD**	**FlsA**	**FlsB**	**FlsC**	**FlsT**	**Accession number**
Salmonella enterica serovar Typhimurium T000240	STMDT12_C39040 to C38980	100	100	100	100	100	100	100	AP011957
Salmonella enterica serovar Enteritidis S14 (pTS14)[b]	Unnamed (16649 to 24218)[c]	100	100	100	100	100	100	100	MN328348
Salmonella enterica serovar Wien ZM3 (pZM3)[b]	NCLADELE_00033 to 00027	100	100	100	100	100	100	100	MK797990
Escherichia coli F11	ECF11_RS19490 to ΔRS19465[d]	99	100	100	100	99	-	-	NZ_AAJU02000030[c]
Yersinia pestis KIM5	CH44_RS16950 to CH44_RS16980[e]	68	88	56	77	73	77	56	NZ_CP009836
Campylobacter jejuni 81-176	CJJ81176_RS07965 to CJJ81176_RS07995[f]	31	55	27	36	30	57	27	NC_008787

[a], Comparisons were performed with the "Amino acids assembled tool" of Clone Manager (CmSuite9), with matches excluding conservative changes.

[b], Like in *S*. Typhimurium LSP 146/02, the genes are plasmid-located in *S*. Enteritidis S14 and *S*. Wien ZM3.

[c], Since the genes are unnamed, the nt positions are indicated.

[d], The accession number corresponds to *E. coli* F11 gcontig_1112495918510, whole genome shotgun sequence. This contig carries the four first genes of the system and a truncated *flsC*-like gene, while the *flsT*-like gene is absent. The first two genes were designated *fetM* and *fetP* by [1].

[e], The genes were termed *fetM*, *fetP*, *flpD*, *flpA*, *flpB*, *flpC*, *flpT* by [2]. *flp* derives from *fet*-linked phenotype, because both *fetP* and the downstream locus were required for growth under iron limiting conditions of a mutant affected in other iron uptake systems (Yfe, Feo and Ybt). Since we do not have evidence for a *fet*-linked phenotype, the *fet*-linked genes of *S*. Typhimurium LSP 146/06 were named *fls* (*fet*-linked *Salmonella*).

[f], The *fetP* gene of *C. jejuni* was termed p19 [3].

1. Koch, D.; Chan, A.C.; Murphy, M.E.; Lilie, H.; Grass, G.; Nies, D.H. Characterization of a dipartite iron uptake system from uropathogenic *Escherichia coli* strain F11. *J Biol Chem* **2011**;*286*(28):25317-30.

2. Fetherston, J.D.; Mier, I.; Truszczynska, H.; Perry, R.D. The Yfe and Feo transporters are involved in microaerobic growth and virulence of *Yersinia pestis* in bubonic plague. *Infect Immun* **2012**;*80*(11):3880-91.

3. Chan, A.C.; Doukov, T.I.; Scofield, M.; Tom-Yew, S.A.; Ramin, A.B.; Mackichan, J.K.; Gaynor, E.C.; Murphy, M.E. Structure and function of P19, a high-affinity iron transporter of the human pathogen *Campylobacter jejuni*. *J Mol Biol* **2010**;*401*(4):590-604.
